# Supplementary material for: Hidden delays of climate mitigation benefits in the race for electric vehicle deployment
Source: Nat Commun. 2023 May 31;14:3164. doi: 10.1038/s41467-023-38182-5 (PMC10232456; doi:10.1038/s41467-023-38182-5)
Supplement: Supplementary file 1 — Supplementary Information [file 41467_2023_38182_MOESM1_ESM.pdf]

Supplementary Information for

Hidden delays of climate mitigation benefits in the race for electric vehicle deployment

Yue Ren[1], Xin Sun[2,3,4], Paul Wolfram[5], Shaoqiong Zhao[1], Xu Tang[1], Yifei Kang[6], Dongchang Zhao[2,3,4], Xinzhu Zheng[1*]

1 School of Economics and Management, China University of Petroleum-Beijing, Beijing 102249, China

2 China Automotive Technology and Research Center Co., Ltd, No. 68, East Xianfeng Road, Dongli District, Tianjin, 300300, China.

3 Automotive Data of China (Tianjin) Co., Ltd., No. 3 Wanhui Road, Zhongbei Town, Xiqing District, Tianjin, 300393, China.

4 Automotive Data of China Co., Ltd., Boxing 6th Road, Beijing Economic Development Zone, Beijing, 100176, China.

5 Joint Global Change Research Institute, Pacific Northwest National Laboratory and University of Maryland, College Park, MD, USA

6 Beijing Yiwei New Energy Vehicles Big Data Application &Technology Research Center, Beijing 100081, China

* Correspondence to xinzhuzheng@cup.edu.cn

This file includes:

Supplementary Tables 1 to 17

Supplementary Figures 1 to 9

Supplementary References

Supplementary Tables 4
Supplementary Table 1. Selected studies on climate mitigation benefits of electric vehicles (EVs) relative to internal combustion engine vehicles (ICEVs). 4
Supplementary Table 2. Selected studies on the greenhouse gas (GHG) emissions break-even points of battery electric vehicles (BEVs). 5
Supplementary Table 3. The percentage of BEVs achieving GBET within the battery warranty period. 6
Supplementary Table 4. Comparison of curb weight and test-based fuel consumption between BEVs and ICEVs. 7
Supplementary Table 5 Size class classification for passenger vehicles 8
Supplementary Table 6. Variables considered in the sensitivity analysis. 9
Supplementary Table 7. Sensitivity coefficients of the influencing factors. 10
Supplementary Table 8. The average greenhouse gas break-even time (GBET) of battery electric vehicles (BEVs) in China by transport mode and size class, compared with internal combustion engine vehicles (ICEVs) in various size classes. 11
Supplementary Table 9. Variables and data sources used in the estimation. 12
Supplementary Table 10. Forecasts of the vehicle kilometers travelled under conservative scenario in 2012-2028 (unit:km). 12
Supplementary Table 11. Forecasts of the vehicle kilometers travelled under radical scenario in 2012-2028 (unit:km). 16
Supplementary Table 12. Greenhouse gas(GHG) emission factors of selected power generation technologies in China from existing studies (unit: gCO_2eq/kWh). 18
Supplementary Table 13. Median Greenhouse gas (GHG) emission intensity of power grids by province in 2012-2028 (unit: gCO_2eq/kWh). 19
Supplementary Table 14. Minimum Greenhouse gas (GHG) emission intensity of power grids by province in 2012-2028 (unit: gCO_2eq/kWh). 21
Supplementary Table 15. Maximum Greenhouse gas (GHG) emission intensity of power grids by province in 2012-2028 (unit: gCO_2eq/kWh). 23
Supplementary Table 16. Data inventory of the China Automotive Life Cycle Assessment Model (CALCM). 25
Supplementary Table 17. Greenhouse gas (GHG) emission factors of materials in CALCD, GREET and Ecoinvent (unit:$kgCO_2e/kg$). 29
Supplementary Figures 31
Supplementary Fig. 1. Average annual mileage distribution of passenger cars in China in 2018. 31
Supplementary Fig. 2. Sales volume and average annual vehicle kilometers traveled (VKT) of battery electric vehicles (BEVs). 31
Supplementary Fig. 3. The greenhouse gas break-even time (GBET) uncertainty using the range approach and orthogonal experimental design (OED) method.. 32
Supplementary Fig. 4. Greenhouse gas break-even time (GBET) of battery electric vehicles (BEVs) compared with various emission-level internal combustion engine vehicles (ICEVs).33
Supplementary Fig. 5. Production volume of battery electric vehicle (BEV) batteries and vehicles by province from 2012-2018. 34
Supplementary Fig. 6. The number of battery electric vehicle (BEV) models per province

relative to the provincial market share of national BEV sales, 2012-2018. 35
Supplementary Fig. 7. Distribution boxplot of the vehicle-model specifications for battery electric vehicles (BEVs) produced and sold in China from 2012 to 2018. 36
Supplementary Fig. 8. Distribution boxplot of the vehicle-model specifications for internal combustion engine vehicles (ICEVs) produced and sold in China from 2012 to 2018. 36
Supplementary Fig. 9. Correlations of vehicle-model specifications between battery electric vehicles (BEVs) and internal combustion engine vehicles (ICEVs). 37
Supplementary References 38

Supplementary Tables

Supplementary Table 1. Selected studies on climate mitigation benefits of electric vehicles (EVs) relative to internal combustion engine vehicles (ICEVs).

Study	Region	System boundary	Study object	GHG type	Mitigation effect (EV relative to ICEV)
Zhou et al.,2013[1]	China	Fuel cycle	BEV	CO_2	35.57%
			PHEV		17.78%
Wu et al.,2018[2]	China	Vehicle+Fuel cycle	BEV	GHG	5.40%
Qiao et al.,2019[3]	China	Vehicle+Fuel cycle	BEV	GHG	18.00%
Zeng et al., 2021[4]	China	Vehicle+Fuel cycle	BEV	GHG	23%
			PHEV		17%
Wu et al.,2012[5]	China	Fuel cycle	HEV	CO_2	29%
			PHEV		50%
			BEV		99%
Moro & Lonza,2018[6]	EU	Fuel cycle	BEV	GHG	26-47%
Ellingsen et al.,2016[7]	EU	Vehicle+Fuel cycle	BEV	GHG	20-27%
Girardi et al.,2015[8]	Italy	Vehicle+Fuel cycle	BEV	GHG	50%
Petrauskienė et al., 2020[9]	Lithuania	Vehicle+Fuel cycle	BEV	CO_2	26-47%
Lajunen et al.,2016[10]	Finland and California	Fuel cycle	BEV	CO_2	75%
Bauer et al.,2015[11]	Global	Vehicle+Fuel cycle	BEV	GHG	50- 80%
			HEV		16%
			FCEV		3%

Note: BEV = Battery Electric Vehicles; PHEV = Plug-in Hybrid Electric Vehicle; FCEV = Fuel Cell Electric Vehicle; HEV = Hybrid Electric Vehicle; ICEV = Internal Combustion Engine Vehicle; GHG - Greenhouse Gas.

Supplementary Table 2. Selected studies on greenhouse gas (GHG) emissions break-even points of electric vehicles (EVs).

**Study**	**Region**	**Scope**	**Key assumptions**			**Life cycle inventory (LCI) database**	**Break-even points**
			**Emission intensity of electricity mix**	**Vehicle lifetime**	**Annual vehicle mileage traveled**		
Ellingsen et al., 2016[7]	Europe	Four differently-sized EVs	521 g CO_2/kWh	12 years	15 000 km	Ecoinvent (Ecoinvent Centre 2010)[12]	44 000-70 000 km
Kim et al., 2010[13]	USA	Aluminum versus high-strength steel light-weighting vehicles	GHG emission factors of the average U.S. grid	11-16 years	11 000 km	U.S. life cycle inventory (LCI) database (NREL 2007)[14]	1-11 years (differ by light-weighting options)
Patterson et al., 2012[15]	Europe	Hybrid vehicles and EVs	UK electricity carbon intensity (594 gCO_2e/kWh)	10 years	20000 km	Patterson et al., 2011[16]	1.6-8.3 years (differ by energy scenarios)
International Council on Clean Transportation (ICCT), 2018[17]	Europe	Battery electric vehicles	European average grid electricity	150000 km	N/A	N/A	2-3 years (differ by carbon intensity of grid electricity)
Ambrose & Kendall, 2016[18]	USA	Plug-in electric vehicles	Archsmith et al., 2015[19]	N/A	N/A	GREET (Argonne National Laboratory 2014)[20]	Approximately 400–1150 charging cycles
This study	China	All passenger BEVs produced and sold in 2012-2018	38-801 g CO_2e/kWh (differ by regional electricity mix)	N/A	678-15927 km (vary by province)	CALCAT (2021)[21,22]	From zero to over eleven years with an average of 4.5 years

Note: N/A indicates that the study didn't report such information.

Supplementary Table 3. The percentage of battery electric vehicles (BEVs) achieving the greenhouse gas break-even time (GBET) within the battery warranty period.

Transport mode	Year	2012	2013	2014	2015	2016	2017	2018
	Threshold	Five-year battery warranty				Eight-year battery warranty		
Car	A00	100%	100%	98%	89%	95%	97%	99%
	A0	98%	99%	97%	93%	100%	100%	99%
	A	78%	98%	69%	38%	72%	89%	86%
	B	-	-	0%	0%	70%	61%	66%
SUV	A0	100%	100%	100%	-	99%	94%	96%
	A	-	-	100%	90%	99%	100%	100%
	B	-	-	-	-	100%	100%	100%
	C	-	-	-	-	-	-	100%
MPV	A0	29%	10%	6%	1%	1%	42%	36%
	A	-	-	-	-	-	82%	96%
	B	-	-	-	-	41%	93%	74%

Note: Cell values in the table denote the percentage of BEVs produced and sold in that year achieving GBET within the battery warranty threshold, i.e., five years required in 2014[23] and eight years required in 2016[24]. Since there is no official requirement before 2014, we assume five years (2014 requirement) for 2012 and 2013. A blank cell means there is no corresponding vehicle data. SUV = Sports Utility Vehicle; MPV = Multi-Purpose Vehicle.

Supplementary Table 4. Comparison of curb weight and test-based fuel consumption between battery electric vehicles (BEVs) and internal combustion engine vehicles (ICEVs).

Transport mode	Size class	Average curb weight		Average test-based fuel consumption		Average battery capacity
		ICEV (kg)	BEV (kg)	ICEV (L/100km)	BEV (kWh/100km)	BEV (kWh)
Car	A00	933	974	6.0	13	22
	A0	1090	1267	7.2	15	24
	A	1277	1581	7.1	15	40
	B	1537	1937	7.8	16	55
SUV	A0	1287	1512	7.0	15	45
	A	1576	1787	8.5	17	47
	B	1829	1788	9.8	15	52
	C	2132	2460	13.1	21	67
MPV	A0	1307	1918	7.2	18	51
	A	1555	1878	7.8	16	55
	B	1909	2302	9.7	23	55

Note: SUV = Sports Utility Vehicle; MPV = Multi-Purpose Vehicle.

Supplementary Table 5. Size class classification of passenger vehicles

		Class A00	Class A0	Class A	Class B	Class C
Car	Wheelbase/mm	<2450	2350-2600	2600-2750	2750-2900	2850-3100
	Length-two compartments/mm	<3750	3750-4400	4200-4700	4700-5000	4950-5150
	Length-trim/mm	<4200	4100-4500	4300-4750		
SUV	Wheelbase/mm		<2650	2600-2750	2750-2900	>2900
	Length/mm		<4350	4350-4750	4700-5000	4950-5150
MPV	Wheelbase/mm		<2800	2800-2900	>2900	
	Length/mm		<4600	4600-4800	>4800	

Note: SUV = Sports Utility Vehicle; MPV = Multi-Purpose Vehicle.

Supplementary Table 6. Description of the variables in the sensitivity analysis.

Grouped variable	Life cycle inventories included	More details
Curb weight	N/A	Refer to Figure S7
Battery capacity		
Battery weight		
Test-based fuel consumption		
Greenhouse gas (GHG) emission factors of the power grid		Refer to Table S13-S15.
Annual vehicle kilometers traveled (VKT)		Refer to Table S10-S11.
GHG emission factors of vehicle material production	Vehicle Components (%)	Refer to Table S16.
	Tire Components (%)	
	Lead-acid Battery Materials Components (%)	
	Fluids Components(%)	
	Material GHG emission factor ($kgCO_2e/kg$)	Refer to Table S17.
GHG emission factors of battery material production	Li-ion Battery Materials Components(%)	Refer to Table S16.
	Material GHG emission factor ($kgCO_2e/kg$)	Refer to Table S17.
Electricity consumption during the vehicle production stage	Electricity (kWh per vehicle)	297-345
	Curb weight (kg)	Refer to Figure S7
Electricity consumption during the battery production stage	Electricity (kWh/kWh)	5.8
	Battery capacity (kWh)	Refer to Figure S7

Supplementary Table 7. Sensitivity coefficients of the influencing factors.

**Influencing factor**	**Life-cycle stage**	**Change in influencing factor**	**Change in GBET**	**Sensitive coefficient**
Curb weight (kg)	Vehicle cycle	5.0%	8.5%	1.7
Vehicle Material greenhouse gas (GHG) Emission Factor ($kgCO_2e/kg$)	Vehicle cycle	5.0%	6.0%	1.2
Battery capacity (kWh)	Vehicle cycle	5.0%	5.5%	1.1
Battery Material GHG Emission Factor ($kgCO_2e/kg$)	Vehicle cycle	5.0%	4.7%	0.9
Vehicle kilometers traveled (km/year)	Fuel cycle	5.0%	4.6%	0.9
GHG emission intensity of power grid ($kgCO_2e/kWh$)	Fuel cycle and Vehicle cycle	5.0%	2.4%	0.5
Battery weight (kg)	Vehicle cycle	5.0%	1.8%	0.4
Test-based fuel consumption (kWh/100km)	Fuel cycle	5.0%	1.6%	0.3
Vehicle Production_Electricity (kWh/kg)	Vehicle cycle	5.0%	0.6%	0.1
Battery Production__Electricity (kWh/kWh)	Vehicle cycle	5.0%	0.6%	0.1

Note: The sensitivity analysis is performed using the one-variable-at-a-time perturbation approach. The sensitivity coefficient refers to the change in the greenhouse gas break-even time (GBET) for every 1% change in the influencing factor.

Supplementary Table 8. The average greenhouse gas break-even time (GBET) of battery electric vehicles (BEVs) in China by transport mode and size class, compared with internal combustion engine vehicles (ICEVs) in various size classes.

Transport mode	Size class	ICEV's size class	2012	2013	2014	2015	2016	2017	2018
Car	A00	-	-	-	-	-	-	-	-
		A00	2.2	3.1	2.3	2.5	3.5	3.3	3.2
		A0	1.2	1.9	1.3	1.5	2.3	2	1.8
	A0	A00	5.6	5.6	5.9	5.9	5.3	5.2	5.3
		A0	4.2	4.1	4.2	4.2	3.8	3.3	3.8
		A	3	2.9	2.9	2.9	2.4	1.9	2.5
	A	A0	6.5	6.2	6.6	8.2	8.7	8	7.6
		A	5.0	4.7	4.9	7.1	7.1	6.4	6.3
		B	3.1	2.8	2.7	4.4	4.4	3.8	3.3
	B	A			10.9	11	10.8	9	9.6
		B	-	-	8.1	9.8	7.8	7.5	7.3
		-			-	-	-	-	-
SUV	A0	A00	-	-	-		-	-	-
		A0	3.7	3.7	3.6	-	5.1	5.7	5.2
		A	1.2	1.7	1.7		3.2	3.7	3.2
	A	A0			5.0	8.2	8.2	7.5	6.4
		A	-	-	2.9	3.2	5.6	5.0	4.3
		B			1.4	1.6	3.5	3.2	2.7
	B	A					5.6	6.2	4.7
		B	-	-	-	-	3.8	4.1	3.1
		C					1.7	1.9	1.3
	C	B							8
		C	-	-	-	-	-	-	4.8
		-							-
MPV	A0	A00	-	-	-	-	-	-	-
		A0	8.6	9.4	10.5	10.9	10.9	7.8	8.4
		A	8.2	8.8	9.4	9.7	9.6	2	4
	A	A0						8.8	7.7
		A	-	-	-	-	-	7.2	6.1
		B						4.3	3.5
	B	A					11	7.6	8.6
		B	-	-	-	-	9.0	5.2	5.8
		C					-	-	-

Note: SUV = Sports Utility Vehicle; MPV = Multi-Purpose Vehicle. Short string indicates insufficient data for the transport mode in that year. The confidence level corresponds with the sample size. Sample size <1000, [1000,10000], >10000 corresponds to low confidence, medium confidence, and high confidence, represented by *, **, and ***, respectively.

Supplementary Table 9. Variables and data sources used in the estimation.

Category	Resolution	Variable	Time	Description	Range	Data source	More details
I	Vehicle level (real-world)	Vehicle sold location	Yearly (2012-2018)	The provinces where vehicles are sold	31 provinces[a]	China's Compulsory Traffic Accident Liability Insurance (CTALI) data, which is provided by China Automotive Technology & Research Center (CATARC)[21,22]	Figure S5-S6.
		Vehicle production location		The provinces where vehicles are produced			
		Battery production location		The provinces where BEV batteries are produced			
		Vehicle model type		Vehicle model types	227 types for BEVs and 1667 types for ICEVs		
II	Vehicle-model level	Curb weight (kg)		The weight of the vehicle, including a full tank of fuel (or battery) and all standard equipment	670-2685 kg for BEVs	Announcement of Vehicle Manufacturing Enterprises and Vehicle Products[25]	Figure S7-S9.
					645-3200 kg for ICEVs		
		Battery capacity (kWh)		Li-ion battery capacity	10.56-78.84 kWh		
		Battery weight (kg)		Battery pack weight	118-750 kg		
		Test-based electricity consumption		Official fuel consumption (electricity or fuel)	9.5-27.9 kWh/100km		

		(kWh/100km)		per 100km based on New European Driving Cycle (NEDC) test condition			
		Test-based fuel consumption (L/100km)			4.6-21.8 L/100km		
III	Provincial level	Vehicle kilometers traveled (km)	Yearly (2012-2030)	Annual mileage traveled by vehicles	678-15927 km/year (2018)	Data for 2018 are obtained from the National Big Data Alliance of New Energy Vehicles (NDANEV)[26]. Data for other years are projected based on our assumptions.	Table S10.
		Greenhouse gas (GHG) emission factors of electricity generation		Annual GHG emissions per kWh of electricity generation	38-801 gCO_2e/kWh (2018)	Data for 2012-2019 are calculated according to the grid structure data from China Electric Power Yearbook 2021[27] and GHG emission factors of power generation technology from IPCC Fifth Assessment Report (AR5) Annex III Table A.III.2[28]. Data for 2020-2028 are projected based on assumptions.	Table S13.
IV	Transport-mode level	Life cycle inventories	Constant	Life cycle inventory at different stages of automobile life cycle assessment	N/A	China Automotive Life Cycle Assessment Model (CALCM)[29]	Table S16.

Note: [a]Data for the Hong Kong Special Administrative Region (SAR), Macao SAR, and Taiwan province are unavailable. BEVs = Battery Electric Vehicles; ICEVs = Internal Combustion Engine Vehicles.

Supplementary Table 10. Forecasts of the vehicle kilometers travelled (VKT) under the conservative scenario in 2012-2028 (unit:km).

**Province**	**2012**	**2013**	**2014**	**2015**	**2016**	**2017**	**2018**	**2019**	**2020**	**2021**	**2022**	**2023**	**2024**	**2025**	**2026**	**2027**	**2028**
Beijing	3333	3547	3774	4015	4272	4546	4837	5252	5703	6193	6725	7302	7929	8610	9350	10153	11025
Tianjin	4545	4836	5145	5475	5825	6198	6595	6978	7384	7814	8269	8750	9259	9798	10368	10971	11610
Hebei	2923	3110	3309	3520	3746	3985	4241	4655	5111	5611	6160	6763	7425	8151	8949	9825	10786
Shanxi	4682	4982	5301	5640	6001	6385	6794	7171	7570	7991	8435	8903	9398	9920	10471	11053	11667
Inner Mongolia	3853	4099	4362	4641	4938	5254	5590	5997	6434	6903	7406	7946	8525	9146	9812	10527	11294
Liaoning	2421	2576	2740	2916	3102	3301	3512	3917	4368	4872	5433	6059	6757	7536	8404	9372	10452
Jilin	1753	1865	1984	2111	2246	2390	2543	2873	3246	3667	4143	4681	5289	5975	6751	7627	8617
Heilongjiang	1847	1965	2091	2225	2367	2519	2680	3037	3441	3899	4417	5005	5671	6426	7281	8249	9347
Shanghai	5852	6227	6625	7049	7500	7980	8491	8798	9116	9445	9787	10140	10506	10886	11279	11687	12109
Jiangsu	5748	6116	6508	6924	7367	7839	8341	8655	8981	9319	9670	10035	10413	10805	11212	11635	12073
Zhejiang	5273	5611	5970	6352	6758	7191	7651	7997	8358	8735	9130	9542	9973	10424	10894	11386	11901
Anhui	3670	3905	4155	4421	4704	5005	5326	5737	6180	6657	7171	7724	8321	8963	9655	10400	11203
Fujian	6877	7317	7785	8283	8813	9377	9978	10322	10679	11048	11430	11825	12234	12657	13094	13546	14015
Jiangxi	1737	1849	1967	2093	2227	2369	2521	2850	3222	3643	4119	4657	5266	5954	6731	7611	8605
Shandong	2447	2604	2770	2948	3136	3337	3551	3956	4408	4912	5473	6098	6794	7570	8435	9398	10471
Henan	2063	2195	2336	2485	2645	2814	2994	3361	3773	4236	4756	5339	5994	6729	7554	8481	9521
Hubei	7767	8264	8793	9356	9954	10591	11269	11541	11819	12104	12396	12695	13001	13315	13636	13965	14302
Hunan	4264	4537	4827	5136	5465	5815	6187	6582	7002	7449	7924	8430	8968	9541	10150	10797	11487
Guangdong	10977	11680	12427	13222	14069	14969	15927	16090	16255	16422	16590	16760	16932	17105	17281	17458	17637
Guangxi	1495	1591	1692	1801	1916	2039	2169	2483	2843	3255	3727	4267	4885	5592	6403	7330	8392
Hainan	4584	4878	5190	5522	5875	6251	6651	7033	7437	7864	8316	8794	9299	9833	10398	10995	11626
Chongqing	2778	2956	3146	3347	3561	3789	4031	4445	4900	5402	5956	6566	7239	7981	8799	9701	10695
Sichuan	8168	8691	9247	9839	10469	11139	11851	12086	12326	12570	12820	13074	13333	13597	13867	14142	14422

Guizhou	5399	5744	6112	6503	6919	7362	7833	8171	8523	8891	9274	9674	10091	10526	10980	11454	11947
Yunnan	1785	1900	2021	2150	2288	2435	2590	2922	3296	3719	4195	4732	5338	6022	6793	7663	8644
Tibet	467	497	529	563	599	637	678	833	1023	1257	1544	1896	2329	2861	3514	4316	5302
Shaanxi	5059	5383	5727	6094	6484	6899	7340	7698	8074	8468	8881	9314	9769	10245	10745	11269	11819
Gansu	6301	6704	7133	7589	8075	8592	9142	9527	9928	10347	10782	11237	11710	12203	12718	13253	13812
Qinghai	6211	6608	7031	7481	7960	8470	9012	9403	9810	10236	10680	11143	11626	12131	12657	13206	13779
Ningxia	2184	2324	2473	2631	2800	2979	3170	3565	4010	4511	5074	5707	6419	7220	8121	9135	10275
Xinjiang	4805	5112	5439	5788	6158	6552	6971	7343	7734	8147	8581	9038	9520	10027	10562	11125	11718

Note: Data for the Hong Kong Special Administrative Region (SAR), Macao SAR, and Taiwan province are unavailable.

Supplementary Table 11. Forecasts of the vehicle kilometers travelled (VKT) under the radical scenario in 2012-2028 (unit:km).

Province	2012	2013	2014	2015	2016	2017	2018	2019	2020	2021	2022	2023	2024	2025	2026	2027	2028
Beijing	3333	3547	3774	4015	4272	4546	4837	7897	9025	9617	10247	10919	11635	12398	13211	14077	15000
Tianjin	4545	4836	5145	5475	5825	6198	6595	9963	10638	11104	11592	12101	12632	13186	13765	14369	15000
Hebei	2923	3110	3309	3520	3746	3985	4241	7356	10668	11132	11617	12122	12650	13200	13775	14374	15000
Shanxi	4682	4982	5301	5640	6001	6385	6794	11906	19469	19535	19601	19667	19733	19799	19866	19933	20000
Inner Mongolia	3853	4099	4362	4641	4938	5254	5590	6718	8165	8810	9506	10257	11067	11941	12884	13902	15000
Liaoning	2421	2576	2740	2916	3102	3301	3512	12182	13101	13324	13552	13783	14018	14257	14501	14748	15000
Jilin	1753	1865	1984	2111	2246	2390	2543	5855	8372	9005	9686	10418	11206	12053	12965	13945	15000
Heilongjiang	1847	1965	2091	2225	2367	2519	2680	7556	10314	10808	11326	11869	12438	13034	13659	14314	15000
Shanghai	5852	6227	6625	7049	7500	7980	8491	14186	13815	13958	14102	14248	14395	14544	14695	14847	15000
Jiangsu	5748	6116	6508	6924	7367	7839	8341	15634	18372	18568	18766	18966	19169	19373	19580	19789	20000
Zhejiang	5273	5611	5970	6352	6758	7191	7651	15626	18236	18447	18662	18878	19098	19319	19544	19770	20000
Anhui	3670	3905	4155	4421	4704	5005	5326	7637	9948	10472	11024	11605	12216	12859	13536	14249	15000
Fujian	6877	7317	7785	8283	8813	9377	9978	25144	28160	28160	28160	28160	28160	28160	28160	28160	28160
Jiangxi	1737	1849	1967	2093	2227	2369	2521	5315	7983	8637	9346	10113	10942	11840	12812	13863	15000
Shandong	2447	2604	2770	2948	3136	3337	3551	6623	9105	9692	10316	10980	11687	12439	13240	14093	15000
Henan	2063	2195	2336	2485	2645	2814	2994	6417	8341	8975	9659	10394	11185	12037	12953	13939	15000
Hubei	7767	8264	8793	9356	9954	10591	11269	14773	19336	19418	19500	19583	19665	19749	19832	19916	20000
Hunan	4264	4537	4827	5136	5465	5815	6187	10722	15876	16341	16820	17312	17819	18341	18878	19431	20000
Guangdong	10977	11680	12427	13222	14069	14969	15927	28674	30223	30223	30223	30223	30223	30223	30223	30223	30223
Guangxi	1495	1591	1692	1801	1916	2039	2169	6994	7556	8232	8969	9772	10646	11599	12637	13768	15000
Hainan	4584	4878	5190	5522	5875	6251	6651	13303	18048	18281	18518	18757	18999	19245	19493	19745	20000
Chongqing	2778	2956	3146	3347	3561	3789	4031	10492	18636	18801	18968	19136	19306	19477	19650	19824	20000
Sichuan	8168	8691	9247	9839	10469	11139	11851	15550	18028	18263	18502	18743	18988	19236	19488	19742	20000

Guizhou	5399	5744	6112	6503	6919	7362	7833	19040	22851	22851	22851	22851	22851	22851	22851	22851	22851
Yunnan	1785	1900	2021	2150	2288	2435	2590	7901	14871	14887	14904	14920	14936	14952	14968	14984	15000
Tibet	467	497	529	563	599	637	678	3801	9139	9723	10344	11005	11708	12456	13252	14099	15000
Shaanxi	5059	5383	5727	6094	6484	6899	7340	19553	34191	34191	34191	34191	34191	34191	34191	34191	34191
Gansu	6301	6704	7133	7589	8075	8592	9142	23044	16966	17319	17678	18046	18421	18803	19194	19593	20000
Qinghai	6211	6608	7031	7481	7960	8470	9012	53555	50539	50539	50539	50539	50539	50539	50539	50539	50539
Ningxia	2184	2324	2473	2631	2800	2979	3170	9487	7677	8347	9076	9869	10731	11668	12687	13795	15000
Xinjiang	4805	5112	5439	5788	6158	6552	6971	18797	23015	23015	23015	23015	23015	23015	23015	23015	23015

Note: Data for the Hong Kong Special Administrative Region (SAR), Macao SAR, and Taiwan province are unavailable.

Supplementary Table 12. Greenhouse gas (GHG) emission factors of selected power generation technologies in China from existing studies (unit: gCO_2eq/kWh).

Power generation technology	Coal-fired power	Hydro power	Wind power	Nuclear power	Photovoltaic power
Ding et al. (2017)[30]	1045	15.5	8.42	6.31	50.2
Wang et al. (2019)[31]	-	3.84	28.3	12.4	-
Li et al. (2019)[32]	744.5	-	31.32	-	-
Gao et al. (2019)[33]	-	-	51.57	-	13.5
Wang et al. (2021)[34]	660-1050	12.4	3-41	3-35	13-190
IPCC (this study)[28]	820	24	11	12	48

Supplementary Table 13. Median greenhouse gas (GHG) emission intensity of power grids by province in 2012-2028 (unit: gCO_2eq/kWh).

Province	2012	2013	2014	2015	2016	2017	2018	2019	2020	2021	2022	2023	2024	2025	2026	2027	2028
Beijing	798	801	798	803	789	786	792	789	680	667	654	641	628	615	605	594	584
Tianjin	813	812	812	811	808	804	801	790	768	729	690	652	613	574	570	566	562
Hebei	772	765	758	752	728	711	698	679	686	656	626	596	567	537	527	517	507
Shanxi	795	790	785	775	756	744	730	722	746	748	751	753	755	758	759	760	762
Inner Mongolia	741	727	728	717	703	695	691	690	724	722	720	718	716	714	727	739	752
Liaoning	742	695	686	675	639	621	592	579	277	233	188	143	98	54	169	283	398
Jilin	678	629	685	690	653	639	630	629	674	595	517	439	360	282	237	192	148
Heilongjiang	753	727	734	738	723	704	687	672	619	547	475	403	332	260	327	394	462
Shanghai	815	813	812	809	806	802	798	796	793	780	767	754	741	728	652	576	501
Jiangsu	778	778	774	770	767	754	730	710	642	662	683	703	724	744	740	736	731
Zhejiang	658	670	662	617	613	627	612	586	393	353	314	274	234	195	297	400	503
Anhui	802	802	798	792	778	769	760	759	749	751	754	756	759	761	643	524	406
Fujian	527	592	564	491	385	435	475	456	437	450	463	476	489	502	512	522	531
Jiangxi	663	703	694	670	653	673	693	664	482	393	304	215	126	37	99	161	223
Shandong	804	799	796	791	788	780	760	729	710	694	678	662	646	630	525	421	316
Henan	780	786	788	781	783	769	744	726	721	730	739	747	756	764	732	700	667
Hubei	330	399	355	372	358	347	378	422	176	181	186	191	195	200	312	424	537
Hunan	526	549	507	475	460	491	535	489	103	90	77	64	50	37	66	95	124
Guangdong	661	628	645	626	585	601	593	550	515	536	557	578	599	620	517	414	311
Guangxi	462	511	426	352	372	364	414	453	255	213	171	130	88	46	149	252	355
Hainan	692	716	702	751	566	530	535	510	123	107	90	74	57	41	67	93	119
Chongqing	514	571	532	533	528	535	569	567	130	121	112	104	95	86	85	84	83
Sichuan	242	204	163	130	106	103	116	123	82	74	66	58	50	43	50	58	66
Guizhou	541	612	493	465	500	502	498	514	641	539	437	335	234	132	124	116	108

Yunnan	236	209	143	106	101	84	95	97	139	141	144	146	148	151	199	248	296
Tibet	175	231	121	24	30	32	38	37	25	25	25	25	25	24	39	53	67
Shaanxi	766	770	768	756	756	723	724	711	671	682	693	704	716	727	587	446	306
Gansu	502	490	492	481	484	443	434	416	673	591	509	428	346	264	331	398	466
Qinghai	186	208	201	194	245	226	146	121	487	401	315	229	143	57	163	268	373
Ningxia	769	756	745	724	687	680	670	670	732	691	650	609	568	527	432	336	241
Xinjiang	697	688	669	666	645	638	640	636	681	655	629	603	576	550	570	589	609

Note: Data for the Hong Kong Special Administrative Region (SAR), Macao SAR, and Taiwan province are unavailable.

Supplementary Table 14. Minimum greenhouse gas (GHG) emission intensity of power grids by province in 2012-2028 (unit: gCO_2eq/kWh).

Province	2012	2013	2014	2015	2016	2017	2018	2019	2020	2021	2022	2023	2024	2025	2026	2027	2028
Beijing	758	761	758	763	749	746	752	750	646	634	621	609	597	584	574	565	555
Tianjin	772	771	771	770	768	764	761	750	729	693	656	619	582	545	542	538	534
Hebei	733	727	720	714	692	676	663	645	651	623	595	567	538	510	501	491	482
Shanxi	755	751	746	736	718	707	693	686	709	711	713	715	718	720	721	722	724
Inner Mongolia	704	691	692	681	668	660	656	655	688	686	684	682	680	679	691	702	714
Liaoning	705	660	652	642	607	590	563	550	263	221	178	136	94	51	160	269	378
Jilin	644	597	650	656	620	607	598	598	640	566	491	417	342	268	225	183	140
Heilongjiang	715	690	697	701	687	668	653	638	588	520	451	383	315	247	311	375	439
Shanghai	774	772	771	769	766	762	758	756	754	741	729	717	704	692	620	548	476
Jiangsu	740	739	735	732	728	716	693	674	610	629	649	668	688	707	703	699	695
Zhejiang	625	637	629	586	582	596	581	556	373	336	298	260	223	185	282	380	477
Anhui	762	762	758	752	739	731	722	721	711	714	716	718	721	723	611	498	386
Fujian	501	563	536	466	365	414	451	433	415	428	440	452	464	477	486	495	505
Jiangxi	630	667	659	636	620	640	659	631	458	373	289	204	120	35	94	153	212
Shandong	764	759	756	751	749	741	722	692	674	659	644	629	614	598	499	400	300
Henan	741	747	749	742	743	730	707	689	685	693	702	710	718	726	695	665	634
Hubei	313	379	338	353	340	330	359	401	168	172	177	181	186	190	297	403	510
Hunan	499	522	481	451	437	466	508	464	98	85	73	60	48	35	63	90	118
Guangdong	628	597	613	595	556	571	564	522	489	509	529	549	569	589	491	393	295
Guangxi	439	485	404	335	353	346	394	430	242	202	163	123	83	44	141	239	337
Hainan	657	681	667	714	538	503	508	485	117	101	86	70	54	39	63	88	113
Chongqing	488	542	506	506	502	508	540	539	123	115	107	98	90	82	81	80	78
Sichuan	230	194	155	124	101	98	110	117	78	71	63	55	48	40	48	55	63
Guizhou	514	582	469	442	475	477	473	488	609	512	415	319	222	125	118	110	102

Yunnan	224	198	136	101	96	80	90	92	132	134	136	139	141	143	189	235	281
Tibet	166	220	114	23	29	30	36	35	24	24	24	23	23	23	37	50	64
Shaanxi	727	731	730	718	718	687	687	675	637	648	659	669	680	691	557	424	290
Gansu	477	466	468	457	460	421	412	395	639	562	484	406	329	251	315	378	442
Qinghai	177	198	191	184	233	214	139	115	463	381	299	218	136	55	155	255	355
Ningxia	730	718	708	688	653	646	637	636	695	656	618	579	540	501	410	320	229
Xinjiang	662	653	635	633	612	606	608	605	647	622	597	572	548	523	541	560	579

Note: Data for the Hong Kong Special Administrative Region (SAR), Macao SAR, and Taiwan province are unavailable.

Supplementary Table 15. Maximum greenhouse gas (GHG) emission intensity of power grids by province in 2012-2028 (unit: gCO_2eq/kWh).

Province	2012	2013	2014	2015	2016	2017	2018	2019	2020	2021	2022	2023	2024	2025	2026	2027	2028
Beijing	1022	1026	1022	1029	1011	1007	1015	1012	886	871	857	843	829	814	803	791	780
Tianjin	1041	1040	1040	1038	1036	1031	1028	1015	990	946	902	859	815	771	767	762	758
Hebei	990	982	974	966	938	918	903	882	887	851	815	779	743	707	695	683	671
Shanxi	1018	1012	1007	994	971	957	941	932	963	966	969	971	974	977	978	980	982
Inner Mongolia	953	935	937	924	907	898	892	891	933	930	928	925	923	921	918	915	912
Liaoning	952	893	882	869	823	802	767	750	369	314	258	202	146	91	92	93	94
Jilin	870	807	879	886	839	824	814	816	868	771	673	576	478	381	459	538	616
Heilongjiang	966	933	943	948	929	906	887	869	798	709	620	530	441	352	374	397	419
Shanghai	1044	1041	1040	1037	1033	1029	1023	1022	1017	1002	986	970	954	938	937	935	934
Jiangsu	998	997	992	988	984	969	939	915	829	854	879	905	930	956	947	937	928
Zhejiang	845	860	850	794	788	808	791	758	510	460	410	360	310	260	254	248	241
Anhui	1027	1027	1021	1014	998	989	979	978	961	964	967	970	973	976	924	872	819
Fujian	673	759	723	631	495	563	614	589	564	581	598	614	631	648	609	570	531
Jiangxi	848	899	888	857	837	865	893	857	624	512	401	289	177	66	83	101	119
Shandong	1030	1024	1020	1014	1011	1002	978	939	918	899	880	861	842	823	817	810	804
Henan	998	1006	1009	1001	1003	987	957	935	930	940	951	961	971	982	981	980	979
Hubei	418	508	451	473	456	443	483	540	231	238	244	250	256	263	260	258	256
Hunan	671	701	647	606	588	628	686	627	140	124	108	92	75	59	77	96	114
Guangdong	848	807	828	805	752	774	765	710	664	691	717	744	771	797	782	767	752
Guangxi	589	651	542	448	475	466	532	582	333	282	230	178	126	74	106	138	171
Hainan	886	918	901	964	730	685	692	664	175	154	134	113	92	71	81	91	101
Chongqing	656	729	679	680	674	683	727	725	172	161	150	139	129	118	117	116	115
Sichuan	305	256	203	161	131	127	144	154	105	95	86	76	66	56	69	82	95
Guizhou	690	783	629	593	639	642	637	658	820	689	558	427	296	164	232	299	366

Yunnan	297	263	179	132	127	106	119	122	178	182	186	189	193	196	183	169	156
Tibet	224	300	160	32	43	48	59	61	31	30	30	29	28	28	28	28	28
Shaanxi	980	986	984	969	970	931	932	917	872	886	900	913	927	941	912	882	853
Gansu	644	631	636	625	629	577	559	545	872	775	678	581	484	387	463	540	617
Qinghai	236	268	266	261	332	310	205	177	636	529	422	315	208	102	99	97	95
Ningxia	987	971	959	934	890	882	870	870	946	898	850	802	754	706	735	763	792
Xinjiang	893	883	862	859	832	825	828	823	883	852	822	791	760	730	713	696	679

Note: Data for the Hong Kong Special Administrative Region (SAR), Macao SAR, and Taiwan province are unavailable.

Supplementary Table 16. Data inventory of the China Automotive Life Cycle Assessment Model (CALCM).

Category	Variable		Definition	Description
Greenhouse gas (GHG) emissions in component material production	Vehicle components	Body system (%)	Percentage of each raw material in the body system	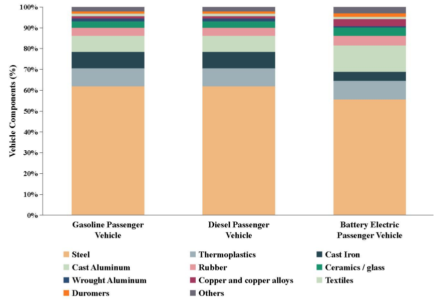
		Powertrain system (%)	Percentage of each raw material in the powertrain system	
		Chassis system (%)	Percentage of each raw material in the chassis system	
		Electric drive system (%)	Percentage of each raw material in the electric drive system	
		Fuel cell onboard storage (%)	Percentage of each raw material in the fuel cell onboard storage	
	Tire components	Rubber (%)	Percentage of rubber in the tire components	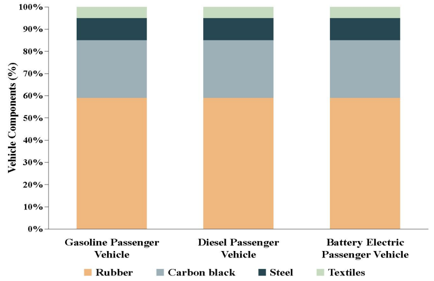
		Carbon black (%)	Percentage of carbon black in the tire components	
		Steel (%)	Percentage of steel in the tire components	
		Textiles (%)	Percentage of textiles in the tire components	
	Lead-acid battery materials	Thermoplastics (%)	Percentage of thermoplastics in the Lead-acid battery materials	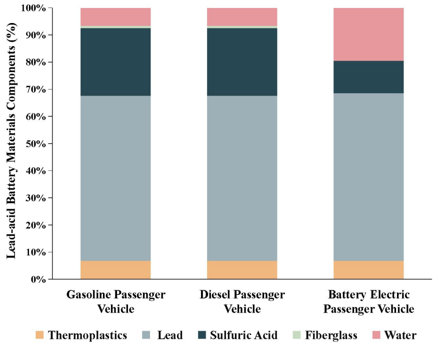
		Lead (%)	Percentage of lead in the Lead-acid battery materials	
		Sulfuric acid (%)	Percentage of sulfuric acid in the Lead-acid battery materials	
		Fiberglass (%)	Percentage of fiberglass in the Lead-acid battery materials	
		Water (%)	Percentage of water in the Lead-acid battery materials	

	Fluids components	Lubricants (%)	Percentage of lubricants in the fluids	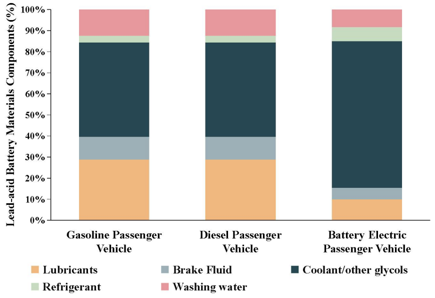
		Brake fluid (%)	Percentage of brake fluid in the fluids	
		Coolant/other glycols (%)	Percentage of coolant/other glycols in the fluids	
		Refrigerant (%)	Percentage of refrigerant in the fluids	
		Washing water (%)	Percentage of washing water in the fluids	
	Li-ion battery materials	NMC (Lithium Nickel Cobalt Manganese Oxide) (%)	Percentage of NMC in the Li-ion battery materials	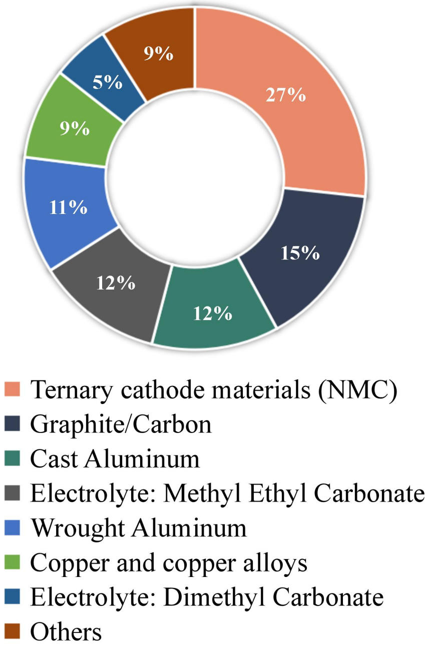
		Graphite/Carbon (%)	Percentage of graphite/carbon in the Li-ion battery materials	
		Electrolyte: LiPF6 (%)	Percentage of electrolyte (LiPF6) in the Li-ion battery materials	
		Electrolyte: ethylene carbonate (%)	Percentage of electrolyte (ethylene carbonate) in the Li-ion battery materials	
		Electrolyte: dimethyl carbonate (%)	Percentage of electrolyte (dimethyl carbonate) in the Li-ion battery materials	
		Electrolyte: methyl ethyl carbonate (%)	Percentage of electrolyte (methyl ethyl carbonate) in the Li-ion battery materials	
		Steel (%)	Percentage of steel in the Li-ion battery materials	
		Wrought aluminum (%)	Percentage of wrought aluminum in the Li-ion battery materials	
		Cast aluminum (%)	Percentage of cast aluminum in the Li-ion battery materials	
		Copper and copper alloys (%)	Percentage of copper and copper alloys in the Li-ion battery materials	

		Thermoplastics (%)	Percentage of thermoplastic in the Li-ion battery materials	
		Printed circuit board (%)	Percentage of printed circuit board in the Li-ion battery materials	
		Coolant / other glycols (%)	Percentage of coolant / other glycols in the Li-ion battery materials	
		Others (%)	Percentage of other materials in the Li-ion battery materials	
	Material production GHG emission factor ($kgCO_2e/kg$)		GHG emissions per kg of raw materials in the production process	Refer to Table S17.
Energy Consumption in Vehicle Production	Stamping, welding, coating, general assembly and power station house	Electricity (kWh/p)	Energy consumption and emissions from various processes in vehicle production stages	297-345 (kWh/p)
		Natural Gas (m^3/p)		12-55 (m^3/p)
		CO_2 ($kgCO_2/p$)		0.6 ($kgCO_2/p$)
		Steam (t/p)		0.3 (t/p)
Replacement	Tires		Times of tire replacements in the life cycle	2
	Battery	Lead-acid battery	Times of Lead-Acid battery replacements in the life cycle	2
		Li-ion power battery	Times of Li-Io battery replacements in the life cycle	0
	Fluids	Lubricants	Times of lubricants replacements in the life cycle	29
		Brake Fluid	Times of brake fluid replacements in the life cycle	2
		Coolant/other glycols	Times of coolant/other glycols replacements in the life cycle	2
		Refrigerant	Times of refrigerant replacements in the life cycle	1
		Washing water	Times of washing water replacements in the life cycle	14
		Natural gas ($kgCO_2e/m3$)	Including natural gas extraction, transportation and other processes	0.07 (kgCO2e/m3)

		Gasoline (kgCO_2e/kg)	Including crude oil fractionation and heavy distillate cracking and other processes	0.65 (kgCO2e/kg)
		Diesel (kgCO_2e/kg)	Including crude oil distillation, catalytic cracking, thermal cracking, hydrocracking, petroleum coking and other processes	0.64 (kgCO_2e/kg)
		Coal (kgCO_2e/kg)	Including mining, screening, coal preparation, coal forming, coal pulping, coal combustion, coal conversion, etc.	0.08 (kgCO_2e/kg)
		Low pressure steam (0.3MPa) (kgCO_2e/kg)	Including raw coal mining, washing process, transportation and boiler steam production process	0.31 (kgCO_2e/kg)
		Medium pressure steam (1MPa) (kgCO_2e/kg)	Including raw coal mining, washing process, transportation and boiler steam production process	0.38 (kgCO_2e/kg)
	Density of fuel	Gasoline density (kg/L)	Weight per liter of gasoline	0.73 (kg/L)
		Diesel density (kg/L)	Weight per liter of diesel	0.85 (kg/L)
Pump to Wheels	GHG emissions factors of fuel use	Gasoline CO2 conversion coefficient (kgCO_2e/L)	GHG emissions per liter of gasoline used	2.37 (kgCO_2e/L)
		Diesel CO2 conversion coefficient (kgCO_2e/L)	GHG emissions per liter of diesel used	2.60 (kgCO_2e/L)

Supplementary Table 17. Greenhouse gas (GHG) emission factors of materials in China Automotive Life Cycle Database (CALCD), Greenhouse gases, Regulated Emissions, and Energy use in Technologies Mode (GREET) and Ecoinvent (unit:kgCO_2e/kg).

CALCD		GREET-US (GREET 2020)		Ecoinvent 3.6-China		Ecoinvent 3.6-Global	
**Variable**	**Value**	**Variable**	**Value**	**Variable**	**Value**	**Variable**	**Value**
Steel	2.38	Average steel (recycled steel production: 26.4%)	2.56	N/A	N/A	GLO[c] market for steel, unalloyed	1.82
						GLO market for steel, low-alloyed	1.58
						GLO market for steel, chromium steel 18/8	4.39
Cast iron	1.82	Mix (final cast iron: 85%)	1.01	N/A	N/A	GLO market for cast iron	1.71
Aluminum and aluminum alloy	16.38	Virgin wrought aluminum<br>Virgin cast aluminum	7.98<br>8.87	N/A	N/A	GLO market for aluminum, cast alloy	5.28
						GLO market for aluminum, wrought alloy	12.7
Magnesium and magnesium alloys	39.55	Virgin magnesium	36.49	CN[b] magnesium production, pidgeon process	30.3	GLO market for magnesium-alloy, AZ91	26.9
Copper and copper alloys	4.23	Mix: copper (chilean copper:15.8%)	2.88	N/A	N/A	GLO market for copper	4.65
Plastics average	4.26	Plastics average	2.47	N/A	N/A	Plastics average	4.05
Lithium nickel cobalt manganate	17.4	NMC[a](622): coprecipitation	19.78	N/A	N/A	N/A	N/A

CALCD		GREET-US (GREET 2020)		Ecoinvent 3.6-China		Ecoinvent 3.6-Global	
**Variable**	**Value**	**Variable**	**Value**	**Variable**	**Value**	**Variable**	**Value**
Graphite	5.48	Graphite	4.81	CN: anode production, graphite, for lithium-ion battery	3.65	GLO market for anode, graphite, for lithium-ion battery	3.66
Electricity	0.635	Distributed - U.S. mix	0.45	CN market group for electricity, high voltage	1.01	N/A	N/A
				CN market group for electricity, medium voltage	1.05		
				CN market group for electricity, low voltage	1.06		
Gasoline	0.651	E10: reformulated gasoline (E10) blending and transportation to refueling station	0.56	N/A	N/A	N/A	N/A
Diesel	0.644	Low-Sulfur diesel from crude oil	0.44	N/A	N/A	GLO market group for diesel	0.486

Note: [a]NMC = Lithium Nickel Cobalt Manganese Oxide; [b]CN = China; [c]GLO = Global.

Supplementary Figures

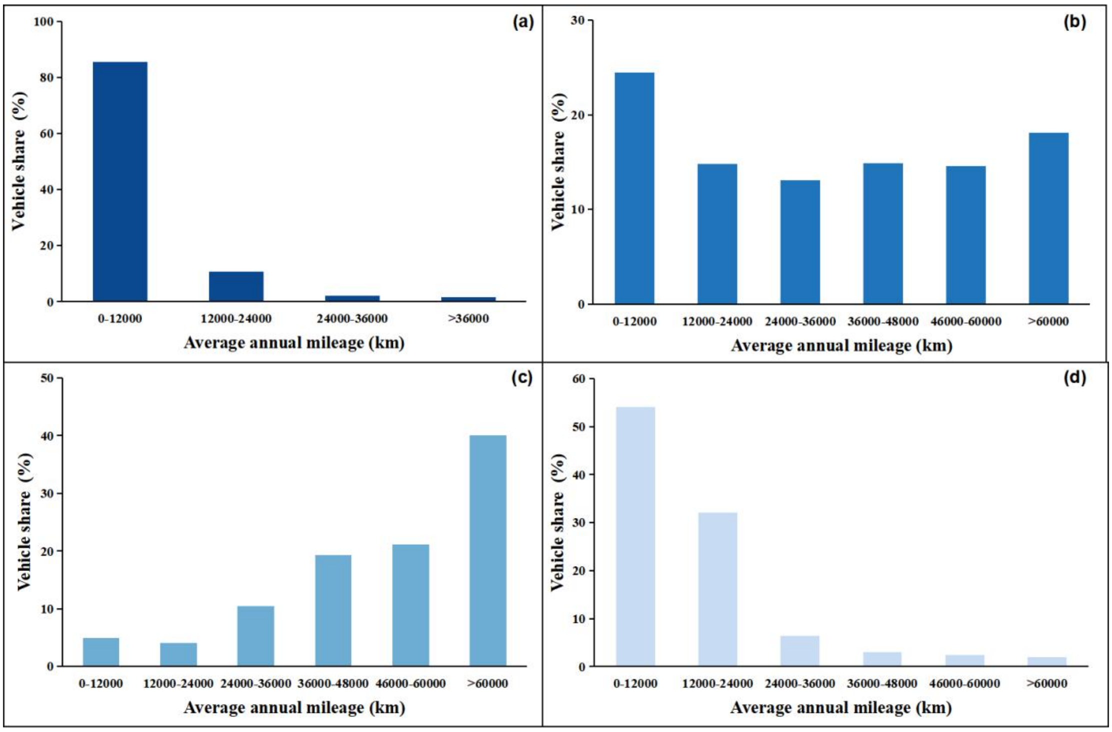

Supplementary Fig. 1. Average annual mileage distribution of passenger cars in China in 2018. a. Private car. **b.** Ride hailing. **c.** Taxicab. **d.** Vehicle leasing.

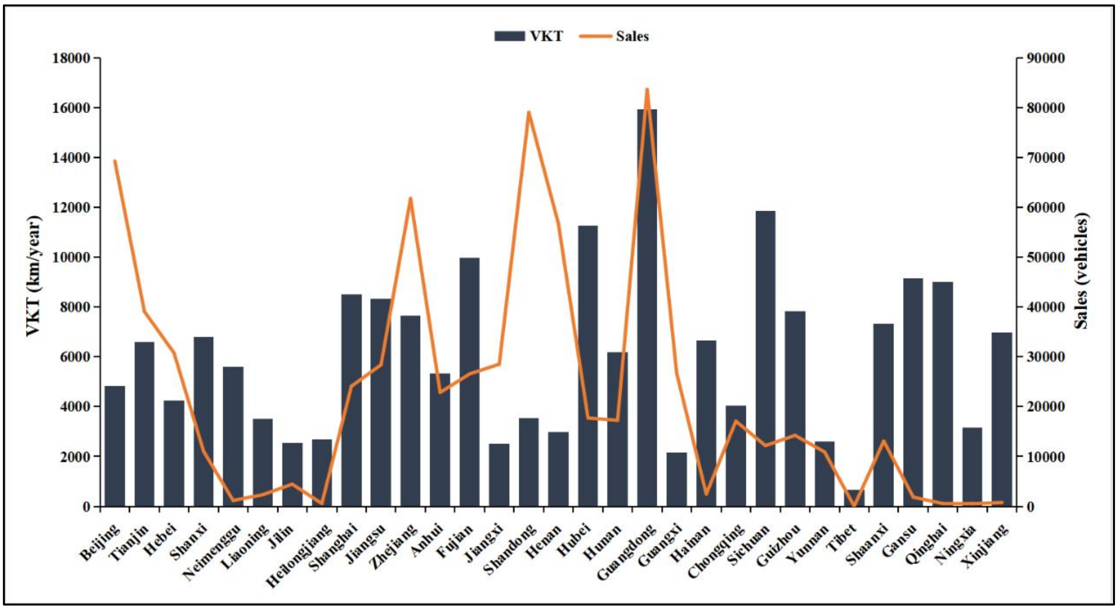

Supplementary Fig. 2. Sales volume and average annual vehicle kilometers traveled (VKT) of battery electric vehicles (BEVs) by province in 2018. Data for the Hong Kong Special Administrative Region (SAR), Macao SAR, and Taiwan province are unavailable.

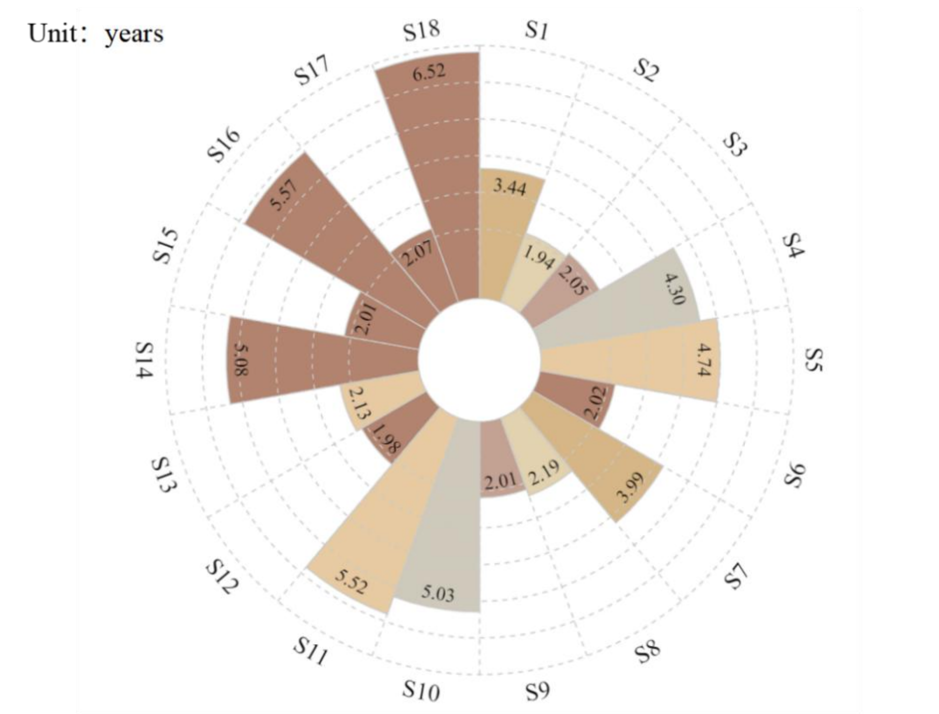

Scenario	Curb weight	Battery capacity	GHG emission factors			VKT
			Vehicle Material	Battery Material	Power grid	
**1**	-5%	-5%	-5%	-5%	Low value	Conservative growth
**2**	-5%	-5%	0	0	High value	Radical growth
**3**	-5%	0	-5%	+5%	High value	Radical growth
**4**	-5%	0	+5%	-5%	Mediumvalue	Conservative growth
**5**	-5%	+5%	0	+5%	Medium value	Conservative growth
**6**	-5%	+5%	+5%	0	Low value	Radical growth
**7**	0	-5%	-5%	+5%	Medium value	Conservative growth
**8**	0	-5%	+5%	-5%	High value	Radical growth
**9**	0	0	0	0	Medium value	Radical growth
**10**	0	0	+5%	+5%	Low value	Conservative growth
**11**	0	+5%	-5%	0	High value	Conservative growth
**12**	0	+5%	0	-5%	Low value	Radical growth
**13**	+5%	-5%	0	+5%	Low value	Radical growth
**14**	+5%	-5%	+5%	0	Medium value	Conservative growth
**15**	+5%	0	-5%	0	Low value	Radical growth
**16**	+5%	0	0	-5%	High value	Conservative growth
**17**	+5%	+5%	-5%	-5%	Medium value	Radical growth
**18**	+5%	+5%	+5%	+5%	High value	Conservative growth

Supplementary Fig. 3. The greenhouse gas break-even time (GBET) uncertainty using the range approach and orthogonal experimental design (OED) method. S1-S18 represent the 18 scenarios under the orthogonal experimental design, and the number of each sector in the chart represents the GBET corresponding to each scenario. GHG = greenhouse gas; VKT = annual vehicle kilometers traveled.

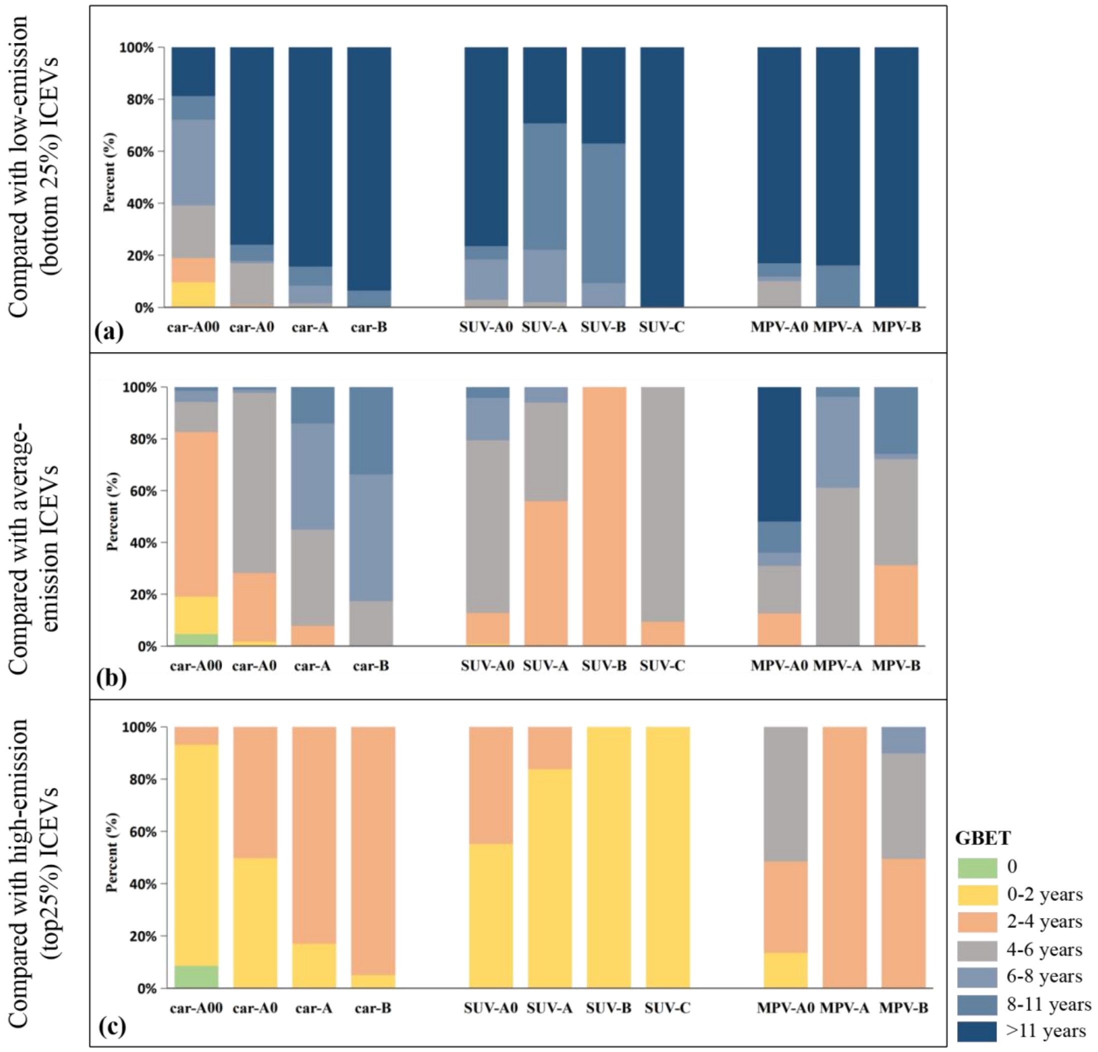

Supplementary Fig. 4. Greenhouse gas break-even time (GBET) of battery electric vehicles (BEVs) compared with internal combustion engine vehicles (ICEVs) at various emission levels. Panel (b) compares all the BEV samples with the average-emission ICEVs in the same vehicle transport mode and size class category car by car. Panel (a) and (c) compare all the BEV samples with the low-emission (bottom 25%) ICEVs and high-emission (top 25%) ICEVs in the same transport mode and size class category, respectively. The bars represent the share of BEVs whose GBET is in a certain range (distinguished by color). SUV = Sports Utility Vehicle; MPV = Multi-Purpose Vehicle.

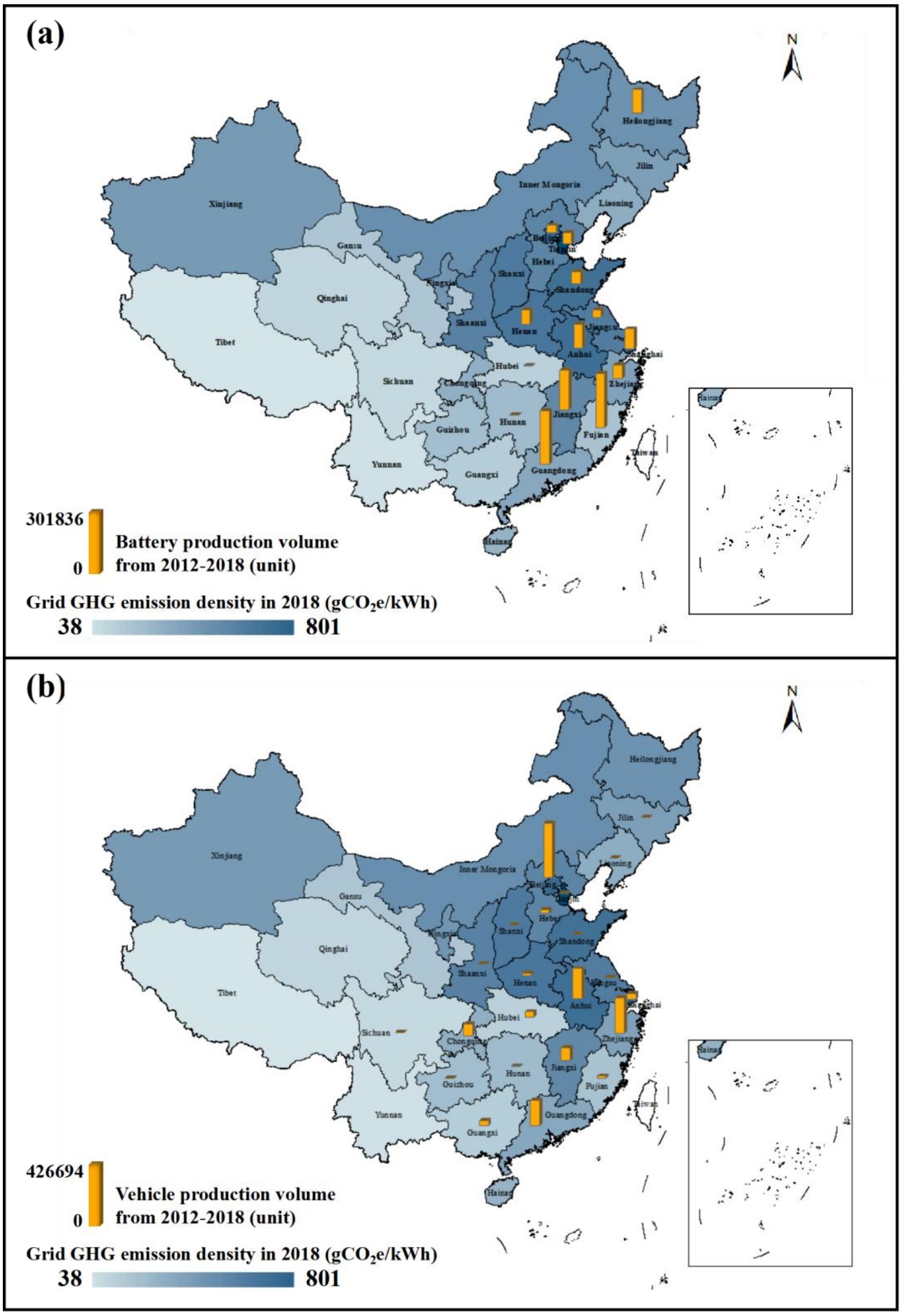

Supplementary Fig. 5. Production volume of battery electric vehicle (BEV) batteries and vehicles by province from 2012-2018. a. Battery production by province. **b.** Vehicle production by province. Data for the Hong Kong Special Administrative Region (SAR), Macao SAR, and Taiwan province are unavailable. GHG = greenhouse gas.

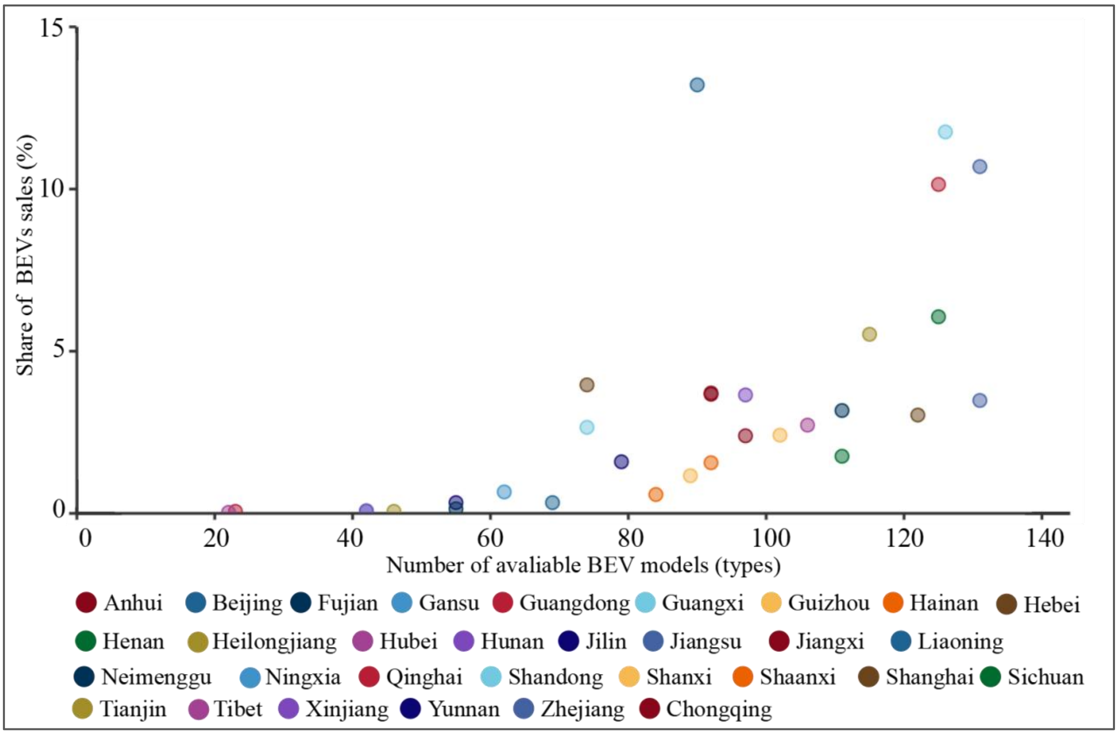

Supplementary Fig. 6. Sales volume Vs. market share of battery electric vehicle (BEV) models per province from 2012 to 2018. The horizontal axis is the number of BEV models sold in Mainland China. The vertical axis is the BEVs market share, which is calculated by dividing the province sales by the national sales. The data for each province is distinguished by color. Data for the Hong Kong Special Administrative Region (SAR), Macao SAR, and Taiwan province are unavailable.

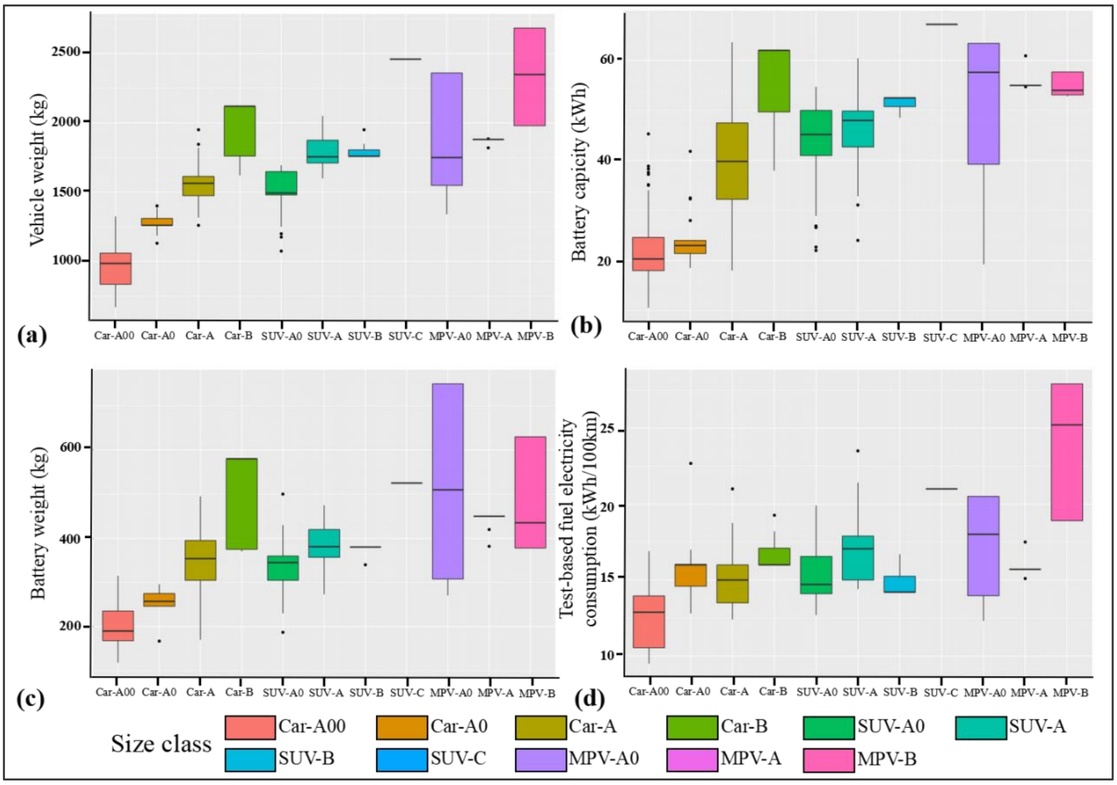

Supplementary Fig. 7. Vehicle-model specification distributions across transport mode and size class for battery electric vehicles (BEVs) produced and sold in China from 2012 to 2018. Panel (a) vehicle weight, (b) battery capacity, (c) battery weight, and (d) test-based electricity consumption.

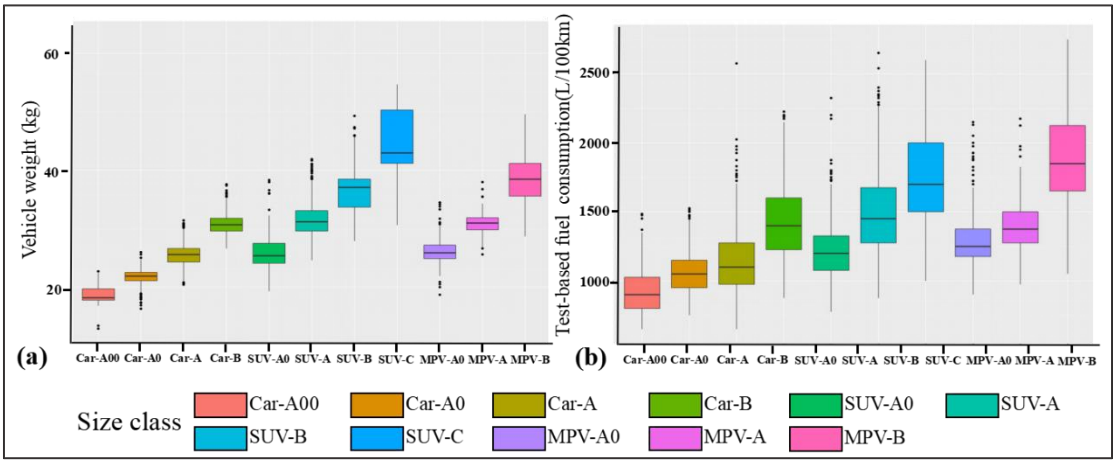

Supplementary Fig. 8. Vehicle-model specification distributions across transport mode and size class for internal combustion engine vehicles (ICEVs) produced and sold in China from 2012 to 2018. Panel (a) vehicle weight, and (b) test-based fuel consumption.

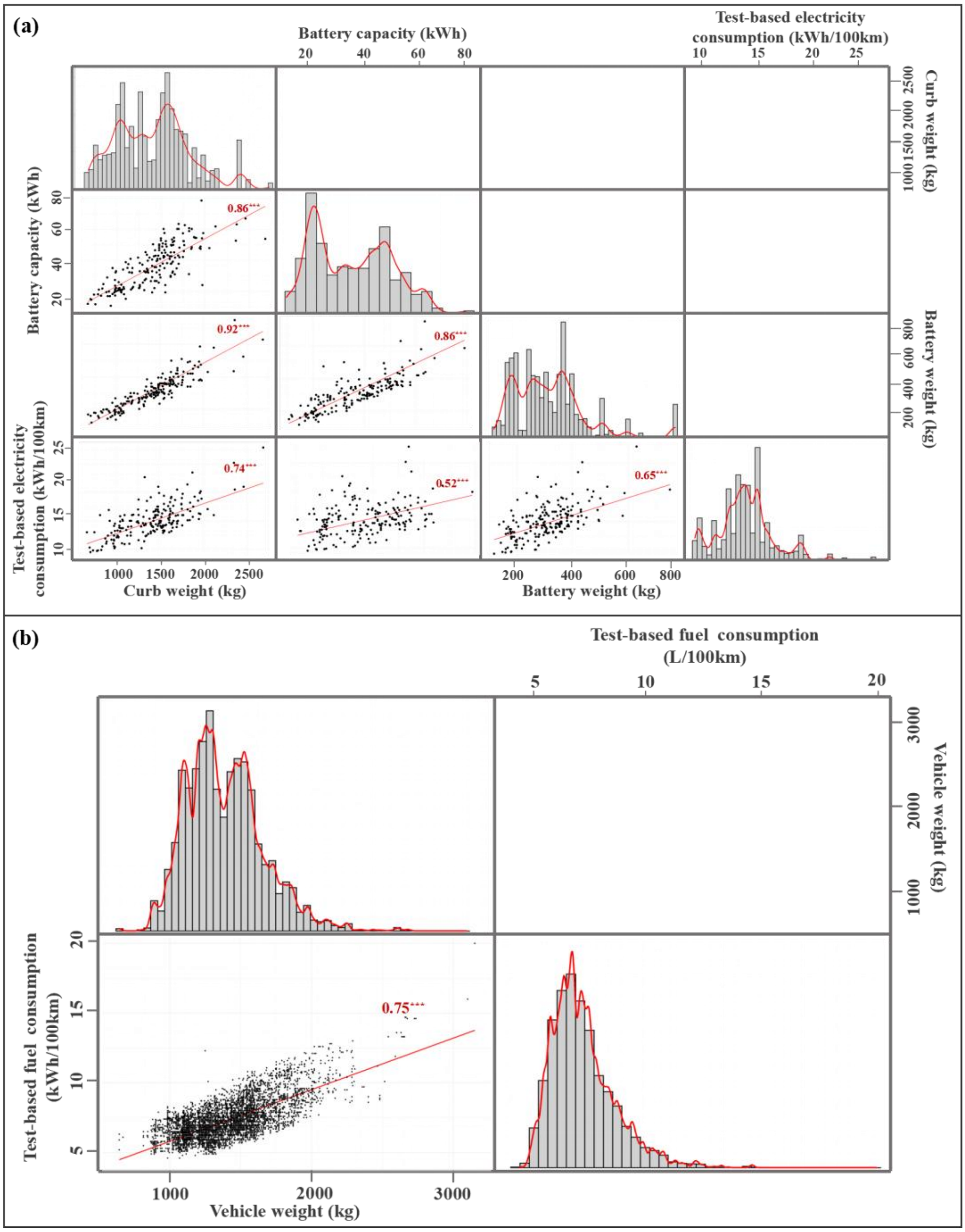

Supplementary Fig. 9. Correlations of different vehicle-model specifications for battery electric vehicles (BEVs) and internal combustion engine vehicles (ICEVs). a. BEV. **b.** ICEV. Panels on the diagonal line show the statistical distribution of each variable. The lower left shows a bivariate scatter plot with fitted lines and correlation coefficients (*** p<0.01, ** p<0.05, * p<0.1).

Supplementary References

1 Zhou, G., Ou, X., & Zhang, X. Development of electric vehicles use in China: A study from the perspective of life-cycle energy consumption and greenhouse gas emissions. *Energy Policy* **59**, 875-884 (2013).

2 Wu, Z. et al. Life cycle greenhouse gas emission reduction potential of battery electric vehicle. *Journal of Cleaner Production* **190**, 462-470 (2018).

3 Qiao, Q., Zhao, F., Liu, Z., He, X. & Hao, H. Life cycle greenhouse gas emissions of Electric Vehicles in China: Combining the vehicle cycle and fuel cycle. *Energy* **177**, 222-233 (2019).

4 Zeng, D. et al. Are the electric vehicles more sustainable than the conventional ones? Influences of the assumptions and modeling approaches in the case of typical cars in China. *Resources, Conservation and Recycling* **167**, 105210 (2021).

5 Wu, Y. et al. Energy consumption and CO_2 emission impacts of vehicle electrification in three developed regions of China. *Energy Policy* **48**, 537-550 (2012).

6 Moro, A. & Lonza, L. Electricity carbon intensity in European Member States: Impacts on GHG emissions of electric vehicles. *Transportation Research Part D: Transport and Environment* **64**, 5-14 (2018).

7 Ellingsen, L. A. W., Singh, B. & Stromman, A. H. The size and range effect: lifecycle greenhouse gas emissions of electric vehicles. *Environmental Research Letters* **11**, 054010 (2016).

8 Girardi, P., Gargiulo, A. & Brambilla, P. C. A comparative LCA of an electric vehicle and an internal combustion engine vehicle using the appropriate power mix: the Italian case study. *International Journal of Life Cycle Assessment* **20**, 1127-1142 (2015).

9 Petrauskiene, K., Skvarnaviciute, M. & Dvarioniene, J. Comparative environmental life cycle assessment of electric and conventional vehicles in Lithuania. *Journal of Cleaner Production* **246**, 119042 (2020).

10 Lajunen, A. & Lipman, T. Lifecycle cost assessment and carbon dioxide emissions of diesel, natural gas, hybrid electric, fuel cell hybrid and electric transit buses. *Energy* **106**, 329-342 (2016).

11 Bauer, C., Hofer, J., Althaus, H. J., Del Duce, A. & Simons, A. The environmental performance of current and future passenger vehicles: Life cycle assessment based on a novel scenario analysis framework. *Applied Energy* **157**, 871-883 (2015).

12 Wernet, G. et al. The ecoinvent database version 3 (part I): overview and methodology. *Int. J. Life Cycle Assess* **21**, 1218–1230 (2016).

13 Kim, H.-J., McMillan, C., Keoleian, G. A. & Skerlos, S. J. Greenhouse Gas Emissions Payback for Lightweighted Vehicles Using Aluminum and High-Strength Steel. *Journal of Industrial Ecology* **14**, 929-946 (2010).

14 National Renewable Energy Laboratory. U.S. Life Cycle Inventory (LCI) Database, https://www.lcacommons.gov/nrel/search (2012). Accessed 5 Aug 2022.

15 Patterson, J., Gurr, A., Marion, F. & Williams, G. Strategic selection of future EV technology based on the carbon payback period. *World Electric Vehicle Journal* **5**, 825-835 (2012).

16 Patterson, J., Alexander, M. & Gurr, A. "Preparing for a life cycle CO_2 measure," Low Carbon Vehicle Partnership, 20 May 2011.

http://lowcvp.org.uk/assets/reports/RD11_124801_4%20-%20LowCVP%20-%20Life%20Cycle%20CO2%20Measure%20-%20Final%20Report.pdf. Accessed 5 Aug 2022.

17 Hall, D. & Lutsey, N. "Effects of battery manufacturing on electric vehicle life-cycle greenhouse gas emissions," The International Council on Clean Transportation, 9 February 2018. https://theicct.org/publication/effects-of-battery-manufacturing-on-electric-vehicle-life-cycle-greenhouse-gas-emissions/. Accessed 5 Aug 2022.

18 Ambrose, H. & Kendall, A. Effects of battery chemistry and performance on the life cycle greenhouse gas intensity of electric mobility. *Transportation Research Part D-Transport and Environment* **47**, 182-194 (2016).

19 Archsmith, J.. Kendall,A. & Rapson, D. From cradle to junkyard: assessing the life cycle greenhouse gas benefits of electric vehicles. *Research in Transportation Economics* **52**, 72-90 (2015).

20 Argonne National Laboratory. The Greenhouse Gases, Regulated Emissions, and Energy Use in Transportation (GREET®) Model. https://greet.es.anl.gov/. Accessed 15 Nov 2022.

21 Auto Data Center of CATARC. http://www.catarc.info/. Accessed 1 Aug 2022.

22 Automotive Data of China Co., Ltd.. China Automotive Low Carbon Action Plan (2022): Low Carbon Development Strategy and Transformation Path for Carbon Neutral Automotive. (China Machine Press, Beijing, 2023).

23 "Announcement on exemption from new energy vehicle purchase tax," State Taxation Bureau of the People's Republic of China, 1 August 2014. http://www.chinatax.gov.cn/n810341/n810755/c1150779/content.html. Accessed 5 Aug 2022.

24 "The financial support for the promotion and application of new energy vehicles during 2016-2020," Ministry of Finance of the People's Republic of China, 29th April 2015. http://www.gov.cn/xinwen/2015-04/29/content_2855040.htm. Accessed 5 Aug 2022.

25 "Automobile announcement inquiry," China Automobile Network. http://chinacar.com.cn/search.html. Accessed 1 Aug 2022.

26 National Monitoring and Management Center for New Energy Vehicles. https://www.evsmc.cn/. Accessed 1 Aug 2022.

27 China Electricity Council. *China Electric Power Yearbook.* (China Statistics Press, Beijing, 2021).

28 Schlömer S. et al. Annex III: Technology-specific cost and performance parameters. *Climate Change 2014: Mitigation of Climate Change. Contribution of Working Group III to the Fifth Assessment Report of the Intergovernmental Panel on Climate Change.* 1329-1356 (2014).

29 "Automotive product life cycle carbon accounting model CALCM-2021 version goes online," Automotive Data of China (Tianjin) Co., L., 23 March 2021. http://www.catarc.info/news/11498.cshtml (2021). Accessed 15 Nov 2022.

30 Ding, N., Liu, J., Yang, J., & Yang, D. Comparative life cycle assessment of regional electricity supplies in China. *Resources, Conservation and Recycling* **119**, 47-59 (2017).

31 Wang, L. et al. A comparative life-cycle assessment of hydro-, nuclear and wind power: A China study. *Applied Energy* **249**, 37-45 (2019).

32 Li, Z. et al. The carbon footprint of large- and mid-scale hydropower in China: Synthesis it from five China's largest hydro-project. *Journal of Environmental Management* **250**, 109363 (2019).

33 Gao, C.-K. et al. Environmental impact analysis of power generation from biomass and wind farms in different locations. *Renewable & Sustainable Energy Reviews* **102**, 307-317 (2019).

34 Wang, M. et al. Study of the emissions and spatial distributions of various power-generation technologies in China. *Journal of Environmental Management* **278**, 111401 (2021).
